# Recreation of a hair follicle regenerative microenvironment: Successes and pitfalls

**DOI:** 10.1002/btm2.10235

**Published:** 2021-06-23

**Authors:** Carla M. Abreu, Alexandra P. Marques

**Affiliations:** ^1^ 3B's Research Group, I3Bs ‐ Research Institute on Biomaterials, Biodegradables and Biomimetics, Headquarters of the European Institute of Excellence on Tissue Engineering and Regenerative Medicine AvePark–Parque de Ciência e Tecnologia, University of Minho Guimarães Portugal; ^2^ ICVS/3B's–PT Government Associate Laboratory Guimarães Portugal

**Keywords:** dermal papilla cells, epithelial cells, extracellular matrix, hair follicle, in vitro models, signaling

## Abstract

The hair follicle (HF) is an exquisite skin appendage endowed with cyclical regenerative capacity; however, de novo follicle formation does not naturally occur. Consequently, patients suffering from extensive skin damage or hair loss are deprived of the HF critical physiological and/or aesthetic functions, severally compromising skin function and the individual's psychosocial well‐being. Translation of regenerative strategies has been prevented by the loss of trichogenic capacity that relevant cell populations undergo in culture and by the lack of suitable human‐based in vitro testing platforms. Here, we provide a comprehensive overview of the major difficulties associated with HF regeneration and the approaches used to overcome these drawbacks. We describe key cellular requirements and discuss the importance of the HF extracellular matrix and associated signaling for HF regeneration. Finally, we summarize the strategies proposed so far to bioengineer human HF or hair‐bearing skin models and disclose future trends for the field.

## 1 THE NEEDS AND DIFFICULTIES OF HUMAN HAIR FOLLICLE REGENERATION

Hair is a characteristic and unique trait of mammals with key physiological functions. Depending on the species and body distribution, the hair follicle (HF) has critical roles in thermoregulation, physical protection, immunosurveillance, relaying sensory perception and as a reservoir of stem cells that are necessary for both homeostasis and response to injury. Moreover, HF has an important aesthetic role for humans, being deeply involved in the individuals' nonverbal communication and well‐being. Thereby, hair loss is invariably associated with a decline in skin function and, depending on the affected area, a profound psychological impact on patients.^1^

The HF is a complex skin appendage that undergoes lifelong cyclical remodeling,^2^ which is reminiscent of the hair embryonic development and equally dependent on different stem cells and well‐orchestrated epithelial–mesenchymal interactions (EMIs).^3^, ^4^, ^5^ However, and unlike what happens in some other mammals, in humans no additional HFs are naturally formed after birth.^6^ When the hair growth machinery is compromised, such as in full‐thickness skin defects or hair disorders, HFs cannot regenerate on their own. In case of extensive injuries, such as severe burns and chronic wounds, the skin loses its regenerative power and autologous grafting is prevented by the shortage of donor skin. Skin substitutes could be considered the ideal treatment, but current products only replicate the epidermal and dermal layers and have limited regenerative capacity, preventing appendages reformation.^1^ Also for hair loss disorders, such as the highly prevalent androgenetic alopecia, there are very few treatments none of which are very effective. Hair growth‐promoting drugs have an unpredictable and limited effect and the only effective treatment requires autologous transplantation of HFs from the healthy occipital area to the affected site, a procedure to which not all patients are electable and that implies the creation of multiple donor sites.

Pioneering studies using rat vibrissa dermal papilla (DP), an inductive mesenchymal structure present at the base of the HF, demonstrated that it was possible to promote HF neogenesis with the transplantation of DP.^7^, ^8^ Ever since the inductive nature of dissociated murine DP (mDP) and dermal sheath (DS, another mesenchymal compartment of the HF) cells was also demonstrated in combination with receptive epithelium cells.^9^ However, it was only after 30 years from the first experiments with murine cells that the inductive nature of intact human DS^10^, ^11^ and DP^12^ was also demonstrated.

While these discoveries have opened possibilities in the development of human HF regenerative strategies, these have been hampered by successive challenges. Comparably to murine, human DP (hDP) cells readily lose their intrinsic inductive properties when removed from their native environment and cultured in vitro.^13^ Indeed, successful human HF regenerative strategies have been hampered by the difficulty in preserving the trichogenic capacity of key mesenchymal and epithelial cells after isolation and/or expansion, both indispensable to obtain clinically relevant cell numbers for biomedical applications.

Along the years, murine models and cells have allowed gaining critical cellular and molecular knowledge on HF embryonic development, postnatal cyclical growth and even regeneration. Nonetheless, there are fundamental interspecies differences that need to be considered. Rodents have different types of hair, some of them highly specialized (e.g., vibrissa), that display a regular distribution pattern and grow in a synchronized way. In contrast, human HFs act independently, have different hair cycle duration and are sensitive to androgens.^14^ Therefore, given the expected low animal‐to‐human translational success rate, human‐dedicated approaches and study platforms in which human HF development, growth and regeneration can be studied remain unfulfilled needs.

**FIGURE 1:**
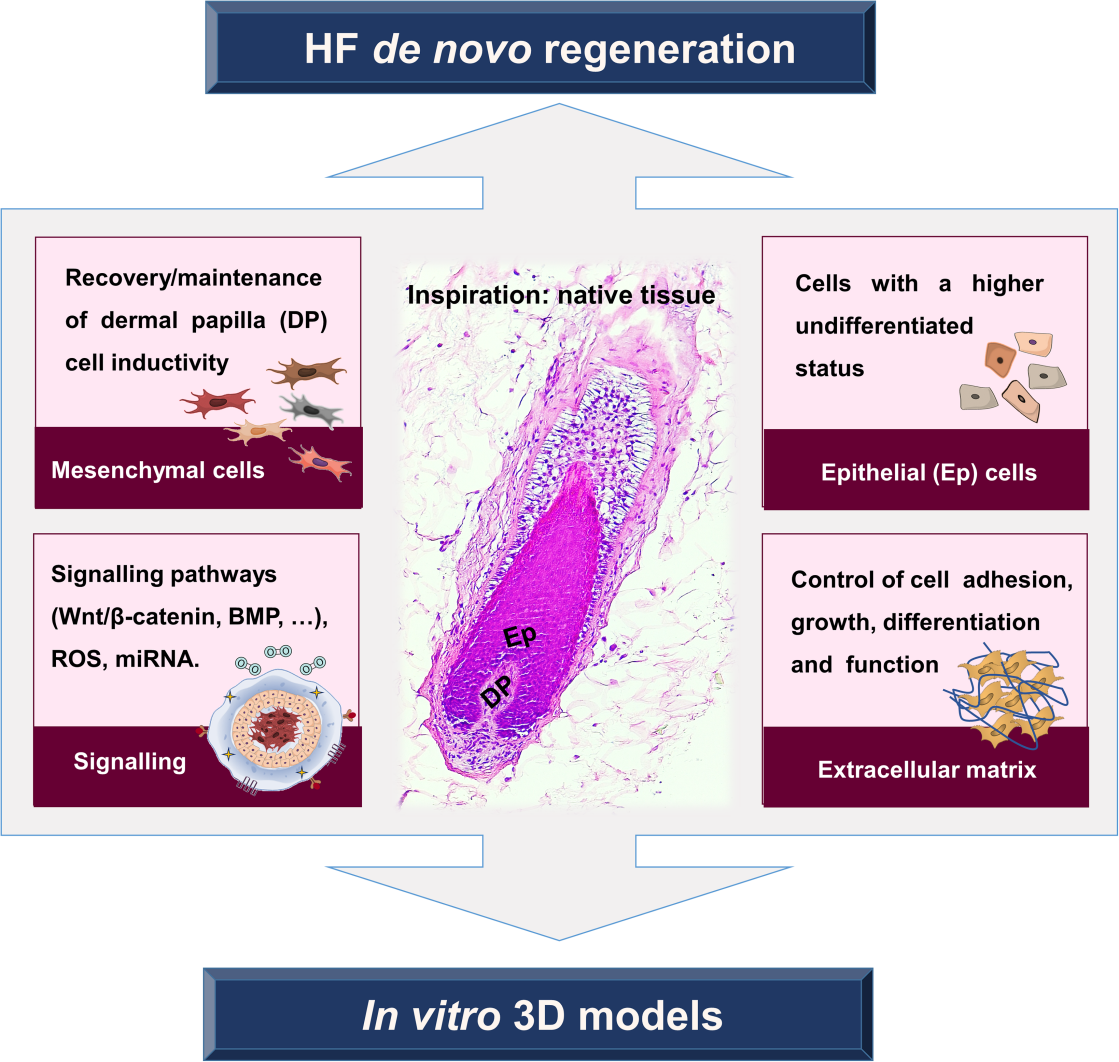
Elements to consider for the recreation of a hair follicle (HF) regenerative microenvironment

This review starts by contextualizing the basic mechanisms underlying HF morphogenesis and cycling and by describing its constituents, including the resident cellular populations, their niches and main functions. Next, relevant aspects associated with the creation of a HF regenerative microenvironment in vitro are discussed. The use of relevant mesenchymal and epithelial cells, the maintenance of their key properties, the importance of a supportive extracellular matrix (ECM) and the existing knowledge of HF‐related signaling events are highlighted (Figure 1). The strategies attempted so far to bioengineer human HF models, or skin models capable of sustaining HF formation in vitro, are then described, emphasizing their achievements and limitations. Finally, general conclusions are drawn and future lines of investigation and important considerations for the field are put in perspective.

## 2 BIOLOGY OF THE HUMAN HF

### 2.1 Embryonic development

**FIGURE 2:**
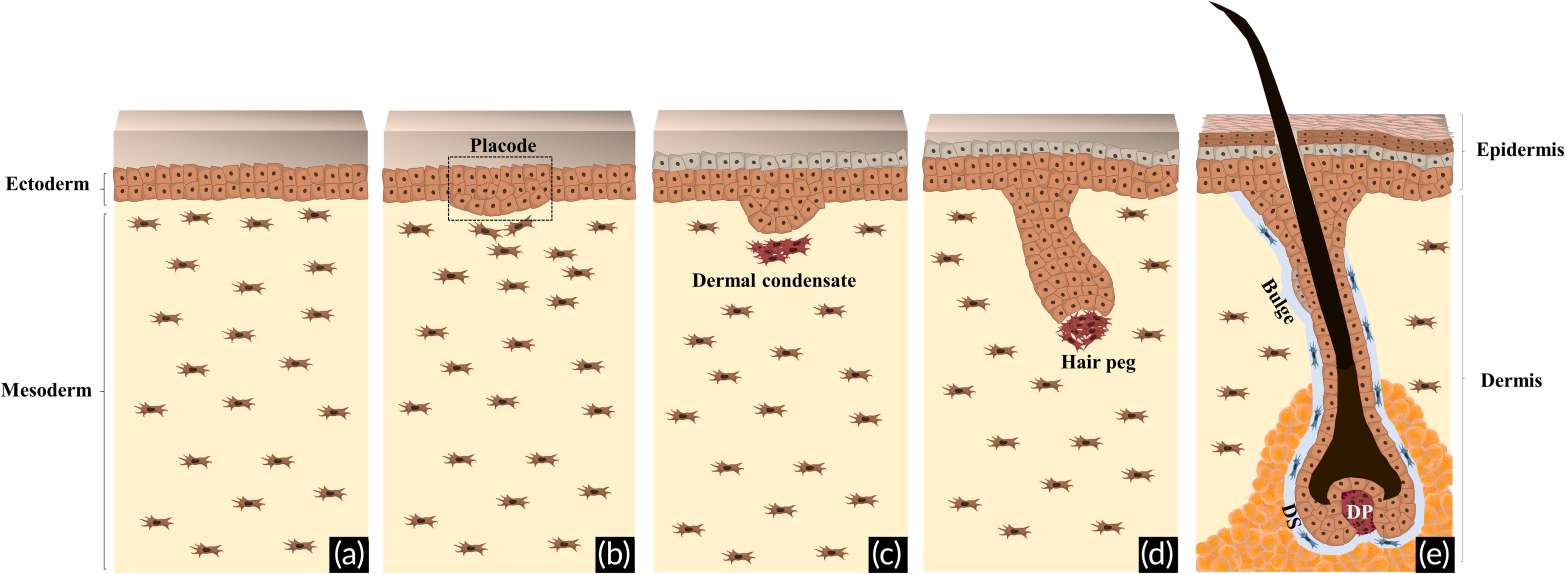
Hair follicle embryonic development. (a) The epithelial and mesenchymal components of the hair follicle (HF) are derived, respectively, from the ectoderm and mesoderm germ layers. (b) Initially, a dermal signal derived from mesodermal cells promotes the formation of spaced thickenings of epidermal progenitors, known as placodes. (c) In response, an epithelial signal stimulates the dermal cells to cluster below the placode, giving rise to the dermal condensate. (d) After a second dermal signal, placode cells start to proliferate and invade the dermis, originating the hair peg. (e) After continuous downward proliferation, the epithelial cells eventually engulf the dermal condensate, which develops into the dermal papilla (DP), allowing the establishment of epithelial–mesenchymal interactions that will further promote the proliferation and differentiation of the epithelial cells into the different structural layers of the mature HF, ultimately leading to the formation of the hair fiber

Depending on their embryonic origin, stem cells can differentiate into the epithelial (ectoderm), mesenchymal (mesoderm) or melanocyte (ectoderm derived neural‐crest cells) lineages that constitute the HF. The HF originates from neuroectodermal–mesodermal interactions (Figure 2(a)), which in humans start by week 12 of the fetal development when a yet unknown signal, considered to arise from the dermis, causes the formation of spaced thickenings in the ectoderm‐derived epithelium, the hair placodes^15^ (Figure 2(b)). At this point, mesenchymal cells located below the stabilized placodes receive epidermal signals to start to aggregate into dermal condensates (Figure 2(c)), establishing the location of the future HF.^16^ Under highly coordinated bidirectional ectoderm–mesoderm interactions, that are triggered by a second dermal signal, the epithelium forms the hair peg (Figure 2(d)) that invades the dermis and eventually surrounds and encloses the dermal condensate. During this process, ectoderm‐derived progenitors give rise to epithelial stem cells (EpSCs) that stay located in the upper part of the incipient HF, in the bulge, providing rapidly dividing cells that allow the downward epithelial movement.^17^ In a parallel process, ectoderm‐derived neural crest cells differentiate into melanocyte stem cells, which will reside in the hair bulge and act as the source of the melanocytes that take part in the pigmentary unit.^2^ By the time epithelial cells engulf the dermal condensate, it differentiates into a specialized mesenchymal population, the DP^3^, ^15^, ^18^ forming, together with the migrating melanocytes, the hair bulb. Along this process, another population of mesoderm‐derived cells surrounds the incipient HF, from the bulge level down to the stalk, bordering the DP and forming the DS, also known as the connective tissue layer. The hair bulb becomes fully mature when it reaches the dermis base and is encased in a layer of dermis‐derived adipose tissue, known as the dermal white adipose tissue (dWAT).^19^ Lineage tracing studies in mice demonstrate that this adipose layer forms independently from the subcutaneous tissue, being originated from a common precursor with the dermal fibroblast lineage, and generates mature adipocytes after HF development.^20^, ^21^ At this point, the DP is fully established as a permanent part of the HF providing the inductive signals necessary to complete HF formation.^15^, ^18^ A well‐controlled epidermal–mesenchymal constant intercommunication (Figure 2(e)) will then be responsible for lifelong hair growth, generating the hair shaft, the part of the HF that is projected beyond the skin and becomes visible on the body surface.

### 2.2 Anatomical components

**FIGURE 3:**
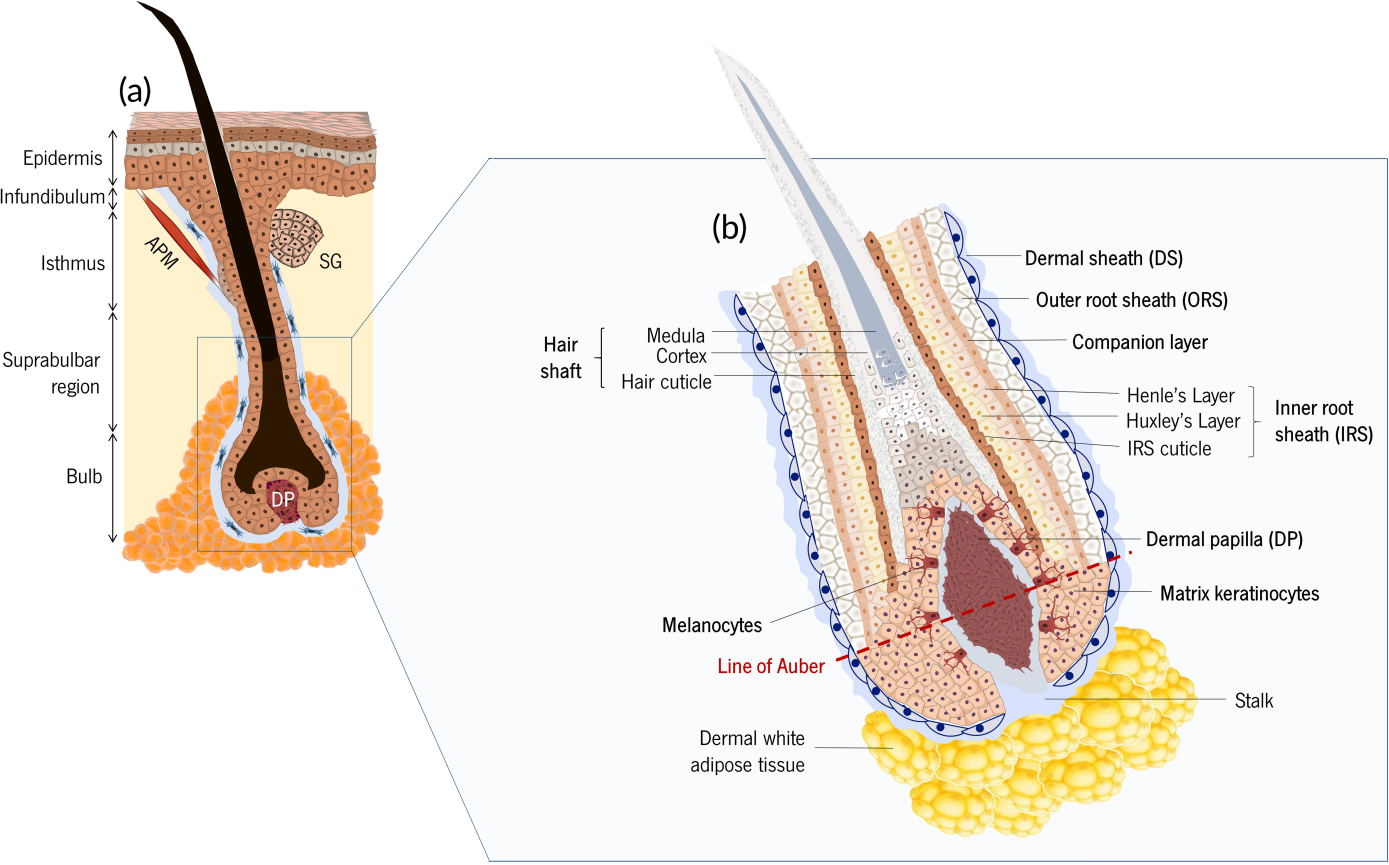
Anatomic representation of the (a) pilosebaceous unit and (b) lower hair follicle

The HF is a singular entity that, together with the sebaceous gland (SG) and arrector pili muscle (APM) forms the pilosebaceous unit^22^ (Figure 3(a)). Anatomically, the HF can be divided into different areas: the infundibulum, isthmus, suprabulbar region and bulb. The infundibulum is in the upper part of the HF, extending from the interfollicular epidermis (IFE) to the sebaceous duct, while the isthmus is located between the SG and the APM, accommodating the bulge. Some literature also refers to the upper part of the isthmus as a separated compartment termed junctional zone.^23^ Below the isthmus lies the suprabulbar area, followed by the hair bulb, the lowest portion of the HF and the one responsible for its active growing.

The hair bulb is divided into two compartments separated by the fictional line of Auber. The DP, the mitotically active epithelial cells that colonize the matrix area (commonly referred to as matrix keratinocytes) and the neural‐crest derived melanocytes are present below this line (Figure 3(b)). The two latter are the respective offspring of keratinocytes and melanocytes progenitors that previously migrated from the bulge through the outer root sheath (ORS), a layer of proliferative epithelial cells that extends down to the hair bulb and divides locally.^24^, ^25^ Above Auber's line lies the offspring of matrix keratinocytes, which differentiated due to the action of factors released by DP,^26^ generating an upward movement through the follicle lumen.

### 2.3 Cycling

Conventionally, a hair growth cycle is divided into three phases: anagen (growing phase), catagen (transition phase), and telogen (resting phase).^18^ During each cycle, the lower part of the HF self‐regenerates, while the upper region, from the bulge level up, remains constant.^18^, ^27^ During anagen, and under highly regulated EMIs, the hair shaft erupts from the skin and the hair fiber grows. In pigmented hairs, this stage is also characterized by the production of melanin by melanocytes and its transfer to the cortical and medullar keratinocytes of the growing hair shaft, making it visible.^28^

After anagen, which in humans can last between 2 and 8 years,^29^ the mature HF entries into catagen. In this destructive phase, generally lasting for 4–6 weeks,^29^ the activity of matrix epithelial cells and melanocytes gradually slows down eventually stopping with the cells entering in an apoptotic state.^2^ As a consequence, the bulb size decreases dramatically and the DP, which was deeply rooted in the dWAT,^19^ regresses and moves upward to the dermis as the remaining epithelial strand regresses. This leads to the formation of the hair germ, constituted by bulge‐derived keratinocytes and other follicular stem cells, localized between the bulge and the DP.^30^, ^31^ At this stage, melanin production ceases,^28^ remaining absent through the 2–3 months^29^ of telogen. Likewise, the DP remains in a quiescent state close to the HF stem cells during telogen.^30^

The shedding of the club hair (telogen hair shaft), initially thought to be a passive process, is now known to be a controlled process and thus considered an independent phase of the hair cycle, the exogen.^32^ After exogen, the HF can initiate a new cycle and regenerate its lower portion by entering in a new anagen phase, which requires the activation of the hair germ.^22^, ^30^ Then, the HF epithelium lengthens downward, pushing down the DP and reestablishing the different epithelial layers. At the end, the new hair bulb, necessary to produce a new hair shaft, is reconstituted.^30^

### 2.4 Stem cell niches

The unique ability of the HF to self‐regenerate cyclically relies on different stem cell populations, located in distinct follicular niches, which coordinate or provide the necessary cellular material for the continuous turnover and replacement of differentiated cells.

The bulge is a particularly privileged structure, comprising multipotent stem cells that can give rise to different HF lineages. In fact, the majority of the clonogenic cells in the HF are derived from this area. EpSCs reside in the bulge as long‐lived quiescent cells^33^ and, as previously mentioned, their offspring is responsible for reconstituting the lower HF apparatus and the derived epithelial layers upon anagen onset.^34^ Moreover, bulge EpSCs can also replenish extrafollicular cellular compartments, including the IFE and SG.^35^, ^36^ Melanocyte stem cells also make their permanent residence in the HF bulge and sub‐bulge region, after migrating from the perinatal dermis and epidermis.^37^ In an analogous way to what happens with EpSCs, melanocyte stem cells give rise to transit‐amplifying melanocyte progenitors, which proliferate and migrate along with the ORS, differentiating into the melanin‐producing melanocytes that during anagen coinhabit the hair bulb with matrix keratinocytes.^37^, ^38^ These die by apoptosis throughout catagen, being afterward replenished by the bulge melanocyte stem cells in the following cycle.^39^ Other neural crest‐like multipotent adult stem cells have been suggested to reside within the bulge, since murine^40^, ^41^ and human^42^, ^43^ cells derived from this area demonstrated the capacity to differentiate in vitro into both neural and mesodermal progeny, including cells from the adipogenic, myofibroblastic, glial, myogenic, and neuronal lineages. Nevertheless, in vivo lineage tracing or functional assays still need to be carried out to confirm the existence and role of these populations in humans.

The bulge is classically considered the HF stem cell reservoir, but during the past decade, evidence collected from murine has demonstrated the presence of epidermal stem cell populations among other HF regions, including the infundibulum, SG, junctional zone, and isthmus.^22^, ^44^, ^45^ Under homeostatic conditions, each subcompartment generally produces a subset of epidermal cells but, upon injury, the different subpopulations can also contribute to a broader range of epidermal lineages.^46^ Although markers of those subpopulations have not been described or confirmed so far in humans, they are most likely contributing to the stem cell heterogeneity in the pilosebaceous unit and maintenance of the different epithelial regions.

Along the hair cycle, the DP suffers volume and cell number fluctuations^47^ while DS regresses during catagen.^48^ Although both DP and DS are known to hold dermal stem cells,^48^, ^49^ DP cells rarely display proliferative features.^47^ Different studies showed that stem cells present in the DS possess the capacity both to regenerate a new DS and supply cells to the DP whenever necessary.^11^, ^48^, ^50^ However, this relation may be more elaborated, since DP cells can themselves contribute to DS renewal during anagen,^51^ suggesting cellular interchangeability and cellular plasticity.^47^ The future identification of distinctive stemness‐related markers and their link to the mesenchymal progeny of the HF might allow to further understand this dynamic and/or potentially identify other critical niches and functions.

## 3 CELLULAR REQUIREMENTS FOR HF REGENERATION

The continuous reconstitution of the HF and its growth machinery after each cycle is dependent on elementary EMIs, implying the need to combine inductive mesenchymal cells and responsive epithelial cells as building blocks in HF regenerative strategies.

### 3.1 Mesenchymal cells

Although the hair mesenchyme is composed of both the DP and DS compartments, DP cells are considered the prime inductive players given their role in the initiation and control of hair formation and growth.^5^, ^52^ The inductive capacity of the peribulbar portion of the DS is sufficient to replace the DP and to regenerate new HFs both in rodents^10^, ^53^ and humans.^11^ However, dissociated rat^54^ DS cells commonly fail to induce hair growth, suggesting that DS‐to‐DP cell transition may be lost upon culture and in the absence of influence from the adjacent epithelium.

The current major challenge associated with the application of hDP cells for HF regenerative purposes is the loss of their inductive phenotype after 2D culture.^13^, ^55^ Therefore, the use of hDP cells for the recreation of the HF microenvironment relies on the recovery and/or maintenance of their native properties in vitro. Up to now, the gold‐standard strategy to preserve hDP cell inductivity is to artificially promote their native intercellular organization and aggregation into 3D spheroids, which partially restores DP cell features^56^ and molecular signature.^13^ Depending on the size of the spheroid, oxygen (O_2_) diffusion might be progressively decreased and whether this play a role in rescuing hDP cell properties in vitro has to be further understood. Cell culture is typically performed under atmospheric O_2_ levels (21% O_2_, normoxia) but in vivo physiological levels usually range between 1 and 11% O_2_.^57^ Comparably to cells cultured under normoxia, hDP cells cultured in 2% O_2_ showed a significant increase in alkaline phosphatase (ALP) activity, a recognized DP cell inductive marker,^50^, ^58^ suggesting improved hair inductive capacity. This was confirmed in vivo, where DP spheroids formed from hDP cells cultured under 2% O_2_ were able to sustain hair neogenesis, unlike cells cultured under atmospheric oxygen levels.^59^

Others have considered DP and bulbar epithelial cells proximity and signaling during anagen, to design new approaches capable of rescuing or preserving native DP cell key features in vitro. We have recently demonstrated that the conditioned medium collected from human skin‐derived keratinocytes (KCs‐CM) improved the inductivity and self‐aggregation capacity of hDP cells, while supporting a secretome profile that matches the one displayed by these cells during anagen.^60^ Moreover, cells cultured with KCs‐CM displayed improved ALP activity compared to hDP cells cultured as spheroids and were capable of reforming HF‐ and SG‐like structures in vivo, suggesting that this could be a superior and simple strategy to improve hDP cell trichogenic capacity.^60^ Interestingly, the supplementation of hDP cells culture with 1α,25‐dihydroxyvitamin D3 (VD3), a compound identified in KCs‐CM, also leads to improved ALP activity. The analysis of possible mechanisms of action revealed that VD3 significantly upregulated Wnt‐10b and TGF‐β2 gene expression in a dose‐dependent manner. However, Wnt inhibition did not hinder VD3 effects on ALP activity. Moreover, as TGF‐β2 upregulation was also maintained and TGF‐β2 signaling was demonstrated to be crucial to hair folliculogenesis and maturation in animal models,^61^ it was suggested that VD3 beneficial effects might involve this growth factor pathway.

Despite these discoveries, strategies that directly target HF signaling pathways known to be downregulated in 2D culture, such as Wnt/β‐catenin, bone morphogenetic protein (BMP) and fibroblast growth factor (FGF)^55^ have been more commonly explored to revert the loss of DP cell inductivity in culture. Adding Wnt‐1a to the culture medium of hDP cells restores the expression of hair induction‐related genes, namely versican, lymphoid enhancer‐binding factor 1 (LEF1), patched homolog‐1, and GLI‐Kruppel family member GLI‐1, previously inhibited with dihidrotestosterone.^62^ Also, human cell treatment with Wnt‐3a and Wnt‐7b enriched extracellular vesicles improved versican, ALP and β‐catenin protein expression and LEF1, vascular endothelial growth factor (VEGF) and keratinocyte growth factor (KGF) transcripts.^63^ An alternative strategy to improve Wnt/β‐catenin activation in hDP cells comprises blocking glycogen synthase kinase‐3, a key enzyme responsible for the degradation of cytoplasmic β‐catenin. So far, molecules successfully used to this end include valproic acid and 6‐bromoindirubin‐3′‐oxime (BIO). Treatment with valproic acid improved ALP activity and quantity in cultured hDP cells, concomitantly with the upregulation of β‐catenin.^64^ In turn, BIO has been mainly used in combination with other biomolecules as a strategy to target multiple signaling pathways. An optimized combination of BIO with recombinant BMP2 and basic FGF (bFGF) maintained the expression of hDP signature genes and improved their ALP activity.^55^ Increased ALP activity and quantity were also confirmed with the supplementation of the culture medium only with BIO and bFGF.^65^ A combined approach showed that VD3, BMP6—a key molecule in maintaining mice DP cell inductive capacity^66^—and valproic acid further enhanced versican expression and ALP activity in comparison to Wnt‐3a treatment.^67^

Another pathway more recently associated with hair growth control is the Janus kinase/Signal transducers and activator of transcription (JAK/STAT), whose pharmacological inhibition has been demonstrated to promote robust hair regrowth in patients with alopecia areata, an autoimmune hair‐loss disorder.^68^ The impact of JAK/STAT inhibitors on the inductive capacity of hDP cells was showed when tofacitinib‐treated hDP spheroids displayed improved HF induction rate than those treated with ruxolitinib, which performed similarly to nontreated spheroids.^69^ The treatment with tofacitinib upregulated TGF‐β2, BMP6, and LEF1 expression^69^ while ruxolitinib improved β‐catenin gene expression but decrease TGF‐β2 expression.^70^ This suggests a TGF‐β2 mediated hair growth after inhibition of the JAK/STAT signaling pathway.

Despite the different rationales and strategies that have been explored so far for the recovery of the inductive phenotype of hDP cells, further studies will be needed to select or develop the most promising one(s).

### 3.2 Epithelial cells

Epithelial cells isolated both from follicular and interfollicular sources possess the capacity to differentiate into follicular epithelium under proper DP cell signaling. Among the follicular sources already described are the bulge,^35^, ^71^ ORS,^72^, ^73^, ^74^ or hair bulb.^75^ Besides, skin epithelial cells have also been successfully combined with DP cells to regenerate HFs, even when isolated from glabrous skin sources such as foreskin.^76^

The differentiation level of the epithelial cells, however, deeply affects their response to inductive dermal cell stimuli and consequently, their trichogenic capacity. K15 positive cells from the skin of adult mice support HF formation when co‐transplanted with neonatal mouse dermal cells, but the same is not observed when the whole epidermal population is used.^35^ This was also demonstrated with human epithelial cells from neonatal skin that, in combination with mDP cells, led to the recreation of a higher number of HF‐like structures than adult skin cells, which lose their trichogenicity after the second passage.^76^ Indeed, few studies demonstrated de novo HF formation from dissociated human cells and most of them report the use of hDP cells with the skin of mouse embryos^77^ or embryonic^78^/newborn^79^, ^80^ skin epithelial cells. To date, entirely human HF regeneration was only succeeded when using epithelial cells from fetal origin^81^, ^82^ or neonatal/children skin.^58^, ^83^ While these keratinocytes display higher competence than adult cells,^74^ the use of newborn cells for regenerative purposes cannot be completely dissociated from ethical concerns. The use of adult cells with stem cell properties, however, can represent a viable alternative. We have recently demonstrated that human IFE epithelial cells with stem‐like features, isolated by fluorescence‐activated cell sorting based on their α6‐integrin^bri^/CD71^dim^ expression, were capable of supporting the formation of immature HFs and SGs when co‐transplanted with hDP cells in mice,^84^ thus representing a promising epithelial cell source for HF regeneration.

Besides preserving epithelial cells undifferentiated state, it is also most advantageous to maintain or promote the trichogenic capacity of the cells in culture. Studies with human cells showed that a follicular‐fate and the trichogenic capacity of keratinocytes can be promoted by coculture with inductive dermal cells. The expression of the epidermal differentiation markers keratin 1 and profillagrin was downregulated in bulge‐derived keratinocytes indirectly cocultured with hDP cells, whereas the expression of the companion layer‐specific markers K75 (formerly known as K6hf) significantly increased, demonstrating that the cells engage in a follicular‐type of differentiation under hDP cell stimuli.^85^ The upregulation of keratins predominantly or exclusively expressed in the HF epithelium, namely K6, K16, K17, and K75, in high passage rat ORS keratinocytes expanded in 3T3 feeders was also reported after coculture with DP cells from the same species.^74^ Moreover, keratinocytes cocultured with DP cells were able to support the formation of new HFs, which was not observed when only clonal expansion in feeders was performed. Similar results were observed when human ORS were grafted with newborn mDP cells.^86^ More recently, follicular keratinocytes cocultured with a CD49f^high^ dermal subpopulation in Matrigel, both from newborn mice, coled the formation of bud‐like structures with an improved expression of Sox9 and ectodysplasin A receptor (Edar), a marker of the developing hair placode.^87^ Nevertheless, in vivo demonstration or translation of those results to human cells has not been performed so far.

Others have shown that the enrichment of mouse IFE cells in the bulge markers integrin α6^+^/CD34^+^ improves cellular trichogenicity, as demonstrated by the increased number of follicles formed after co‐grafting with freshly isolated neonatal mouse fibroblasts.^88^

Overall, the literature demonstrates that different epithelial cell sources may be used for HF regeneration but those with higher undifferentiated status and clonogenic capacity exhibit improved hair regenerative efficiency. Moreover, maintaining in culture the characteristic signaling receptiveness of follicular keratinocytes, or predisposing nonfollicular undifferentiated cells toward a follicular fate, may prove beneficial in improving trichogenic capacity of epithelial cells in culture.

## 4 HF‐RELATED SIGNALING

The events regulating human hair morphogenesis and cycling are still largely unknown. However, it is well‐established that HF formation and growth involves different conserved signaling pathways including, but not limited to, Wnt/β‐catenin, Sonic hedgehog (Shh)/PDGF‐A, FGF, BMP, TGF‐β, and Edar/nuclear factor‐kappa B (NF‐κB) pathways. Although the dissection of the mechanisms regulating HF is beyond the scope of this review (for more details please see References 3, 4, 5, 15, and 89), the knowledge generated so far regarding each one of these signaling pathways, may present opportunities for HF regenerative strategies.

The Wnt/β‐catenin signaling pathway is highly involved in hair development, growth and cycling.^90^, ^91^ Studies in mice showed that the activation of this pathway, and β‐catenin stabilization, direct epidermal cells toward a follicular phenotype.^92^, ^93^ Transient β‐catenin activation in mice epidermis promotes HF neogenesis and induces anagen in follicles in the telogen phase, by improving cell proliferation and outgrowth both from the epidermis and existing ORS.^93^ Wnt/β‐catenin signaling is also critical in the de novo HF formation during wound healing, which reproduces the HF embryonic development.^92^ In vitro, β‐catenin activation and nuclear translocation were linked to the upregulated expression of follicular‐specific epithelial markers in keratinocytes cocultured with DP cells, both for human^85^ and rat cells.^74^ Thus, the role of different Wnt proteins in promoting epithelial cell differentiation has been addressed. Recombinant Wnt‐10b, for example, was shown to be necessary to initiate HF development in mouse embryonic skin organ cultures. Moreover, hair formation was observed only when mice neonatal epithelial cells and fibroblasts were co‐grafted with a fibroblast cell line transfected to produce Wnt‐10b.^94^ In vitro, Wnt‐10b supplementation, contrarily to Wnt‐3a, Wnt‐5a, and Wnt‐11, promoted advanced follicular differentiation of primary keratinocytes from mice skin, suppressing their proliferation and inducing the expression of late differentiation markers, including cortex (mHa5 and mHb5)^95^ and IRS (AE13 and AE15)^96^ specific markers. Moreover, as previously mentioned, Wnt ligands also seem to maintain hDP cell inductivity in vitro. Thereby, Wnt ligands may be used as a strategy to direct keratinocytes follicular differentiation and preserve the identity of DP cells in vitro.

NF‐κB activation through Eda (ectodysplasin)/Edar signaling is also acknowledged as crucial for HF morphogenesis, namely for placode formation, stabilization, and patterning.^97^, ^98^, ^99^ Eda‐A1 is expressed in the epidermis and hair placodes whereas Eda‐A2 is only expressed after, in the hair bulb epithelium.^100^ Probably for this reason, Eda‐A1 seems to be required for ectodermal differentiation, as shown by the inhibition of hair development after mutation of the Eda‐A1 isoform and its receptor.^101^ Nevertheless, both Eda‐A1 and Eda‐A2 isoforms are capable of enhancing the number of epidermal invaginations in skin organ cultures, despite the superior effect of Eda‐A2.^100^ Conversely, postnatal Eda signaling is linked to HF cycle control, particularly in promoting catagen onset and progression.^97^, ^102^ Exploration of possible mechanisms in vitro demonstrated that Eda‐A2 stimulates apoptosis in cultured human ORS keratinocytes and hDP cells,^102^ and that Eda‐A2 receptor is upregulated in balding hDP cells compared to cells from the corresponding nonbalding areas.^103^ Although the role of Eda/Edar signaling has been mainly explored in rodents, its capacity to trigger HF morphogenesis might prove advantageous if explored for human regenerative strategies.

In the mature HF, the activation of BMP signaling is crucial to promote HF differentiation and maturation,^104^ but it needs to be initially counteracted for anagen entry.^105^ Noggin, a protein that neutralizes BMP, is exclusively produced in the HF mesenchyme and knockout experiments in mice showed it to be necessary to stimulate postnatal hair induction.^106^ On the other hand, transgenic mice expressing noggin are characterized by excessive proliferation of matrix keratinocytes and prevention of hair shaft maturation,^104^ clearly demonstrating that a tight spatiotemporal control between BMPs and their inhibitors need to be carefully achieved for their effective use in the recreation of functional hairs.

PDGF‐A and Shh are required to promote DP development and HF maturation.^107^, ^108^, ^109^ PDGF‐A is expressed in mouse embryonic epidermis and developing HF epithelium and acts directly in receptors present in the underlying mesenchyme, promoting their proliferation and the normal development of all skin mesenchymal compartments: DP, DS, and dermis.^107^ Thus, PDGF‐A deficiency results in dermal hypoplasia and impaired DP and DS formation, with animals exhibiting a scarce and thinner hair coat. Shh is required to drive DP formation from dermal condensates, the reason why its deficiency blocks hair formation in mice at an early stage.^107^, ^108^, ^109^ Postnatal PDGF‐A ligands are still required to maintain DP cell function and receptor deficiency results in a progressive depletion of the HF dermal stem cell pool^110^ and disturbs both dermal connective tissue development and hair canal formation.^111^ Also, local injection of PDGF ligands in mice skin induces anagen, which was associated with an upregulation of Shh, Wnt‐5a, and LEF1 at the injection sites.^112^ From the TGF‐β family, only the TGF‐β2 isoform is indispensable to promote hair development, playing a specific role in hair morphogenesis induction that is not shared with other isoforms.^113^ TGF‐β2 treatment in mouse embryonic skin significantly increased the number of hair buds and their stage of development, whereas TGF‐β1 reduced it.^113^ In vitro, TGF‐β2 secretion by hDP cells significantly increases when these are cultured with KCs‐CM, which is associated with an improvement of these cells' inductive capacity.^61^ Indeed, when TGF‐β2 signal was inhibited in mice, HF formation and maturation were significantly impaired.^61^ TGF‐β1, in turn, acts as a negative regulator of keratinocytes proliferation, promoting instead their differentiation and even apoptosis, being commonly appointed as a catagen regulator in murine.^114^ Considering this evidence, it is reasonable to conclude that both PDGF‐A/Shh and TGF‐β2 hold great potential to be used in HF regenerative strategies.

Different elements of the FGF family can also have quite distinct hair regulatory effects. FGF7, also known as KGF, is an important mitogenic factor for keratinocytes and is normally secreted within the DP, contributing to the proliferation of the hair germ cells and activation of a new hair cycle in mice. Its expression increases in telogen but, on its own, is not enough to promote anagen.^30^ In turn, topical application of FGF1, FGF2, and FGF10 in telogenic mice was shown to promote hair growth by inducing early anagen and prolonging its duration.^115^ A similar role was observed for FGF18, whose subcutaneous injection in mice uncovered a potential role in promoting telogen‐to‐anagen transition.^116^ Contrariwise, FGF5 regulates catagen onset in human HF organ cultures. Mutations in this growth factor are associated with increased HF lengthening in humans.^117^ In vitro studies demonstrated that FGF2 increases the proliferation of both hDP and DS cells^118^ and, interestingly, the addition of FGF2 to terminally differentiated human epidermal keratinocytes promoted their dedifferentiation toward an early proliferative.^119^ This increase in the proliferative capacity of both cell types might explain their capacity to induce anagen. Besides the modulation of hair‐related pathways, there are other signaling molecules known to impact hair development and growth. Although classically associated with oxidative stress, reactive oxygen species (ROS) at physiological levels play an important role in hair formation. Bulbar epithelial cells produce high amounts of ROS during hair growing^120^ and studies using human ex vivo HFs organ cultures proved that transient endogenous ROS production promotes HF stem cell activation and anagen entry, which was associated with the activation of Wnt transcriptional factors.^121^ Corroborating these observations, Hamanaka et al.^122^ showed that mice genetically manipulated to prevent mitochondrial ROS generation in basal epithelium displayed impaired development of epidermis, HF and SG. This suggests that both the modulation of different FGFs and the transitory increase of ROS production may represent alternative strategies to promote anagen and hair growth. Yet, studies demonstrating their influence or possible impact during HF development or regeneration are lacking.

MicroRNA (miRNA), involved in the posttranscriptional genetic control of multiple biological processes, also play a role in controlling HF organogenesis and postnatal growth (reviewed in Reference 123). For example, miRNA‐203 targets human and mouse p63, a transcriptional factor highly expressed in the innermost basal keratinocytes, and its upregulation is associated with a decrease of the clonogenic capacity of human keratinocytes.^124^ This explains why its expression is intensified in differentiating cells of the HF.^125^ Similarly, the antiproliferative miRNA‐24 was shown to block the keratinocyte stemness regulator transcription factor 3, compromising the normal process of HF differentiation in mice.^126^ In turn, miRNA‐125b is implicated in stem cells maintenance and its sustained expression, in engineered transgenic mice, blocks hair growth by repressing hair stem cell differentiation.^127^ Noteworthy, miRNA‐31 is associated with HF cycle progression, increasing during anagen and decreasing afterward, and administration of an antisense inhibition was associated with hair shaft defects and ORS hyperplasia in mice.^128^ Exploration of the underlying mechanisms showed that miRNA‐31 acted by targeting the expression of structural keratins in keratinocytes.^128^ Although the incorporation of relevant miRNA in HF regenerative strategies is still theoretical, increased understanding of the involved mechanisms is expected to take miRNA‐based strategies closer to control HF cell behavior.

## 5 THE IMPORTANCE OF THE ECM

In addition to the critical homotypic or heterotypic cell–cell communication, cell–ECM interactions in the HF are crucial not only to maintain the specific cell phenotype and role within the individual compartments but are also involved in physiological (hair cycle) and pathophysiological (hair associated disorders) environment alterations.^129^ The epithelial and mesenchymal compartments of the human HF are separated by a highly specialized ECM basement membrane rich in collagen IV, fibronectin, and laminin.^130^ The hDP ECM also has a prominent expression of these basement membrane elements, comparably to the nonfollicular dermis, along with collagen III, collagen I, versican, and glycosaminoglycans.^131^, ^132^, ^133^

The basement membrane ECM is particularly relevant for epithelial cells differentiation state. Like in the skin, HF basal keratinocytes engagement in a differentiation pathway is associated with the loosening of their interaction with the basement membrane, while the maintenance of the epidermal reservoir is ensured by anchored stem cells.^134^ ORS keratinocytes interact with the basement membrane via α2β1, α3β1, and α6β4 integrins, which are differentially expressed along the HF epithelium, most likely reflecting their differentiation along the HF.^135^ Collagen XVII (COL17A1) is highly expressed in bulge EpSCs and represents a specific stem‐cell anchoring protein required to maintain epithelial and melanocyte stem cell quiescence.^136^ COL17A1‐null mice suffer from premature hair loss and hair graying, which is associated with bulge depletion of EpSCs and also of melanocytes stem cells, which do not express COL17A1 but directly adhere to EpSCs, being equally lost in the process. In humans, mutations in this protein lead to atrophic hair loss associated with generalized atrophic benign epidermolysis bullosa.^137^

The HF ECM is also a key player in the regulation of HF development, hair cycle and signaling. The disruption of the DP ECM and the basal lamina by dispase, a fibronectinase, and Type IV collagenase, inhibited bulb growth in rat vibrissae ex vivo cultures. This confirms the importance of the ECM in supporting hair growth and that the paracrine signaling between epithelial and mesenchymal compartments is not sufficient.^138^ Laminin‐511 is prominently expressed in the basement membrane of the hair germ. Its blockage in embryonic human scalp xenografts induces alopecia, suggesting a role in hair development.^139^ Indeed, mice lacking laminin‐511 have impaired hair development, contained fewer hair germs and exhibit a defective basement membrane compared to wild type, features that can be reverted with exogenous laminin‐511 treatment.^139^ Moreover, laminin‐511 is required for the expression of noggin and downstream regulators, which are necessary for mDP maturation during HF development.^140^

Decorin, expressed in the HF epithelium and ECM of the DP, has been suggested to play a role in anagen maintenance.^141^, ^142^ This is confirmed by the higher expression of this proteoglycan during the anagen phase, and by the accelerated anagen onset in follicles at telogen phase after injection of human decorin in mice, and delayed transition to catagen in those at the anagen stage.^142^ Moreover, decorin‐knockout mice showed shortened anagen, as well as downregulated β‐catenin signaling. In vitro results showed that upregulation of β‐catenin in ORS cells overexpressing decorin occurs together with LEF1 and Wnt‐10b.^141^ This effect was also associated with increased proliferation and migration of human ORS keratinocytes in culture.^141^

A similar impact could be attributed to hyaluronic acid owing to the enhanced ORS growth observed in a human hair germ model in vitro.^67^ Hyaluronic acid, fibronectin, and the proteoglycans aggrecan and biglycan stimulate hDP cell proliferation in vitro.^67^ Further, the motility of rat vibrissae DP cells is diminished on collagen IV, laminin, and collagen I coated surfaces,^143^ while human melanocytes migration is enhanced on collagen IV, but not on fibronectin or laminin.^144^ Interestingly, both RAD16‐I peptide,^145^ a synthetic peptide that shares structural similarity with the integrin receptor‐binding site RGD present in several ECM proteins,^146^ and collagen type IA^78^ hydrogels improved ALP activity and versican expression in hDP cells, suggesting an effect on their inductive potency by these ECM‐replacing matrices in vitro. In turn, the ECM derived from hDP cells supported enhanced tyrosinase activity in melanocytes,^147^ a rate‐limiting enzyme for melanin production. This effect was higher than the one observed in melanocytes cultured onto an hDP cell feeder layer or with hDP cell‐CM, further demonstrating the vital role of the ECM that other signaling mediators cannot replace.

ECM molecules can also sequester and act as a repository of soluble growth factors, protecting them from degradation and regulating their activity and presentation to adjacent cells.^148^ Although no explicit reports are studying or exploring this specific capacity within the human HF, studies performed with ECM elements known to be present in the HF may provide relevant clues. Fibronectin, for example, has a high affinity to growth factors of the VEGF, PDGF and FGF families and, to a lesser extent, to TGF‐β.^149^ Likewise, fibrin, vitronectin, collagens, and proteoglycans are capable of differently bind VEGFs, BMPs, hepatocyte growth factors (HGFs) and TGF‐β.^148^, ^149^ As previously discussed, PDGFs, TGF‐βs, and FGFs have important roles in the control of HF development and/or hair growth. Moreover, VEGF is also required for the reformation of the perifollicular vascularization upon anagen entry^150^ and HGF, a potent mitogenic factor produced by DP cells, stimulates bulbar epithelial cells proliferation.^151^ The ECM can regulate the distribution of growth factors either in the presence of cell‐mediated forces by the action of enzymes, such as metalloproteinases, allowing a spatiotemporal control of growth factor release,^148^ and empowering the ECM with regulatory capacity.

Altogether, these studies highlight the importance of the HF ECM components in balancing epithelial cell growth and differentiation, modulating HF development and EMIs, and influencing cell‐specific function and properties, including the inductive properties of DP cells.

## 6 DEVELOPMENTS IN THE BIOENGINEERING OF HUMAN HF MODELS

Unquestionably, the development of HF regenerative strategies would benefit from the existence of reliable and representative in vitro models allowing, in a standardized way, to test new bioactive compounds to modulate hair growth or to study the biological and molecular events regulating hair morphogenesis and growth.

Over the years, the development of in vitro models of the human HF was looked at from different perspectives. From simple systems where 3D cultured epithelial cells and DP cells were assembled in close proximity, to models that mimicked the spatial organization of the cells within the HF bulb and, more recently, to more elaborate platforms in which HF recreation was viewed not as an isolated structure but instead integrated with the skin. Table 1 summarizes the HF models developed so far using cells of human origin, from the simplest to the more complex, highlighting their features (in vitro) and HF regeneration ability (in vivo).

**TABLE 1:** Reported bioengineered human HF or hair‐bearing skin in vitro models

In vitro structures	Strategy	In vitro experimental findings	In vivo outcomes	Ref.
Epidermoid cyst‐like spheroids 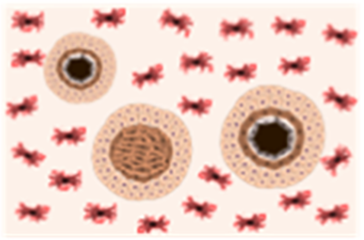	DP cells were: cocultured with ORS or IFE keratinocytes inside Matrigel and layered above cell‐free collagen I gels; embedded in collagen I hydrogels and overlaid with IFE or ORS cells in Matrigel.	Independently of their location, DP cells accelerated epithelial cells proliferation. Both epithelial cell types formed aggregates (larger in ORS cells) but these displayed an epidermis‐type of stratification toward the center instead of a follicle‐type of development.	NA	^152^
“Folliculoid sandwiches” 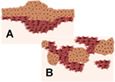	Dermal fibroblasts were mixed with collagen I and covered on top by: A) Matrigel network with DP cells, where ORS cells were seeded on top (layered system) B) A Matrigel network were both DP cells and ORS were encapsulated (mixed system).	The cells remained in close contact and ORS keratinocytes were capable of proliferating and forming epithelial aggregates rather than generating an epidermis‐like stratified epithelia.	NA	^153^
HF‐like structures 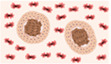	DP or DS cells were encapsulated in a collagen I gel and different epithelial cells—IFE keratinocytes, ORS keratinocytes (superior or inferior anatomical location) or matrix keratinocytes—were seeded on top.	The combination of both ORS and DS cells led the formation of structures with inward oriented epithelial concentric layers, which was not reported for DP cells.	Simple HF‐like structures mainly observed when DP cells and superior ORS keratinocytes were co‐transplanted.	^72^
Folliculoid microsphere 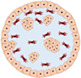	DP cells and ORS keratinocytes were encapsulated in a matrix mixture of collagen I and Matrigel (4:1 ratio) and casted as small droplets.	Small aggregates of DP cells expressing versican or ORS cells positively staining for K6 and displaying proliferative properties were observed, but a HF type of organization was not observed.	NA	^154^
Follicular DP structures 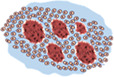	DP cells and IFE keratinocytes were encapsulated in different domains of water‐soluble chitin (polycation), separated by a sodium alginate solution (polyanion) and brought together to form an insoluble fibrous hydrogel by interfacial polyelectrolyte complexation.	DP cells self‐assembled into aggregates, while the adjacent epithelial layers remained in close contact, but only partially surrounding the DP‐spheroids.	Formation of rudimentary HF‐like structures.	^155^
Unpigmentated fiber‐producing structures 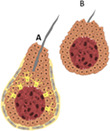	A) DP aggregates were coated with basement membrane proteins (collagen IV, laminin and fibronectin) and ORS keratinocytes and melanocytes were added to the culture afterward.	Formation of organoids in which DP cells were surrounded by concentric epithelial layers, including a K15 and a trichohyalin‐positive layers. Melanocytes remained in close proximity with DP cells and the formation of unpigmented hair‐like fibers was observed.	NA	^73^
	B) DP cells spheroids were formed on top of Matrigel and matrix keratinocytes were posteriorly added.	Formation of structures capable of generating a colorless hair‐like fiber.	NA	^156^
HF organoid model 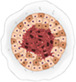	DP‐spheroids were encapsulated in a silk‐gelatin hydrogel and cocultured with both HF stem cells and HF keratinocytes. In a biomaterial‐free approach, DP‐spheroids were directly cultured with both epithelial cells.	Formation of an in vitro HF‐organoid model in which cellular proliferation and the expression of DP cell signature genes versican (ALP, BMP4, and β‐catenin) were significantly higher with the hydrogel support.	NA	^157^
Tubular structures 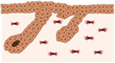	DP cells were embedded in collagen I hydrogels and IFE keratinocytes seeded on top. Alternatively, DP cells were replaced by their conditioned medium in the culture.	Both DP cells and their conditioned medium induced the projection of keratinocytes into the collagen matrix, forming tubular structures that resembled hair germ formation during embryogenesis.	NA	^158^
Epidermal invaginations or folliculoid structures 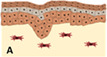	A) DP cells were cultured in porcine acellular dermal matrices and follicular keratinocytes enriched in stem cells were posteriorly seeded on the opposite side.	Constructs with DP cells lead to a better stratified epidermis with a higher number of proliferative basal cells and epidermal invaginations.	Presence of embryonic hair bud‐like structures which expressed the companion layer marker K6hf, demonstrating commitment to the follicular lineage.	^159^
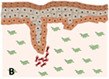	B) DP spheroids were incorporated between layers of fibroblast‐embedded collagen I hydrogels, which were then used to produce standard reconstructed skin.	DP spheroids within dermal equivalents stimulated epidermal downward movement and dermis invasion. These epidermal invaginations were composed of an inner K15‐positive layer, an outer K10‐positive layer and displayed a basement membrane rich in collagen IV and laminin.	NA	^160^
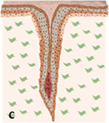	C) DP spheroids surrounded by IFE keratinocytes were embedded in collagen‐based dermal equivalents and microscopy‐guided laser ablation was used to create a hole from the surface of the construct up to the incorporated multicellular aggregates.	The created channel guided keratinocytes migration and downward movement toward the aggregates with DP cells, which led the formation of folliculoid structures that recapitulated the HF microarchitecture.	NA	^161^
HF generation within reconstructed skin 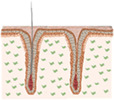	3D‐printed molds were used to produce dermal equivalents with microwells of controlled dimensions, inside which DP cells (transfected or not with an LEF1 vector) spontaneously formed spheroids. Neonatal keratinocytes were then seeded over the dermal constructs.	The keratinocytes encased the DP cell‐spheroids, forming an epithelial column that initiated HF‐specific differentiation, expressing hair lineage markers and occasionally forming colorless hair fibers. DP cells overexpressing LEF‐1 synergistically improved HF differentiation and hair shaft formation. Dermal vascularization was observed in the constructs used for in vivo implantation.	Vascularized constructs induced the formation of unpigmented hairs, but DS reformation in the grafts was not observed.	^83^
Hair‐bearing skin organoid 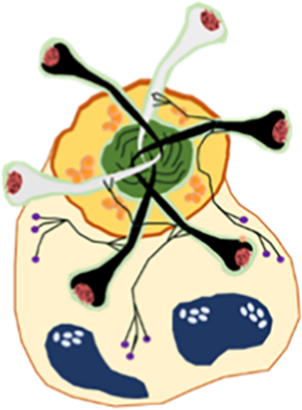	Human embryonic (WA25) or induced pluripotent (DSP–GFP) stem cell lines were used to form aggregates and cultured with 2% Matrigel, bFGF, BMP4, and a TGF‐β inhibitor. Afterward, a BMP inhibitor and bFGF were added to the culture. The resultant organoid structures were culture and matured over 140 days.	Independently of the cell line used organoids acquired a bipolar organization, with a chondral tail and an epidermal cyst head, which developed in complete stratified skin with HFs. The recreated HFs featured all the cellular layers (except the medullar layer), were embedded in a lipid‐rich dermis, in which hairs were associated with SGs and a neuronal network. The number of HFs was higher in WA25 organoids, while a higher number of pigmented hairs were observed in the DSP–GFP organoids.	Organoids without the cartilaginous tail were engrafted in nude mice. In 55% of the xenografts, hair shaft growing perpendicular to the skin surface was observed.	^162^

Abbreviations: ALP, alkaline phosphatase; bFGF, basic FGF; BMP, bone morphogenetic protein; DP, dermal papilla; DS, dermal sheath; FGF, fibroblast growth factor; HF, hair follicle; IFE, interfollicular epidermis; LEF1, lymphoid enhancer‐binding factor 1; ORS, outer root sheath.

The first attempts to construct these HF models were focused on creating 3D systems where the close contact between epithelial and DP cells was reestablished. These constructs depicted simplified spatial organizations and were elementary in what concerns the recapitulation of the HF structure. They involved the coculture of the cells in Matrigel, collagen I or other matrices with different spatial arrangements.^72^, ^152^, ^153^, ^154^, ^155^ Despite the recreation of the signaling in these systems, the epithelial cells often formed cyst‐like spheroidal structures and a follicular‐type differentiation was not observed or confirmed. Therefore, their functionality was short and their use might be restricted to testing simple features, such as the effect of hair growth modulatory agents in epithelial cell proliferation or the expression of specific markers expression, more closely resembling coculture systems than in vitro models of HF.

More complex approaches allowed to create in vitro models in which unpigmented hair fiber‐like structures were formed. In one method DP, spheroids formed on top of Matrigel were directly cocultured with matrix keratinocytes^156^ while, in a slightly more elaborated strategy, DP cell aggregates were coated with basement membrane molecules (collagen IV, laminin, fibronectin) and only then cultured with matrix keratinocytes and melanocytes.^73^ The formation of “microfollicles” with a proper topological cellular arrangement in these models represented a substantial improvement in the fulfillment of criteria steps toward HF formation. In another endeavor, a simple in vitro DP organoid model, characterized by enhanced expression of inductive markers and epithelial proliferation, was developed.^157^ Here, DP spheroids were encapsulated in silk‐gelatin hydrogels together with normal HF keratinocytes and HF stem cells, to mimic the HF bulb cellular environment.

Instead of bioengineering HFs as a single independent structure, others have developed or incorporated HFs in skin constructs, better reproducing the native tissue and, thereby, improving the model functionality and translational value. In a first approach, DP cells' paracrine signaling was explored for the promotion of epidermal keratinocytes organization and formation of tubular epidermal invaginations in an organotypic skin model. Both DP cell‐ and fibroblast‐laden collagen matrices were permissive to this effect although the latter still required the presence of DP cells‐conditioned medium in the culture.^158^ Similarly, epithelial invasion of the dermis was demonstrated when DP cells and follicular keratinocytes enriched in stem cells were seeded in a porcine acellular dermal matrix.^159^ Also, a more robust epidermal protrusion directed toward DP cells was observed when DP spheroids were incorporated within the dermal equivalents of a standard organotypic skin model.^160^ While epithelial invagination and formation of an epithelial column are required for HF morphogenesis, DP spheroids rapidly dissociate when placed within collagen matrices,^83^ which might be compromising DP cell inductivity and model progression. Bearing this in mind, we were able to create a more complex skin model with folliculoid appendages by incorporating, within the dermal equivalent layers, aggregates with compartmentalized DP cells and keratinocytes.^161^ Microscopy‐guided laser ablation was used to fabricate a channel connecting the constructs' surface and the aggregates, to support epithelial cell migration. Follicle‐like structures with differenced concentric epithelial layers and mimicking the hair bulb architecture were obtained, though a hair shaft was not formed. In another strategy where DP spheroids integrity was maintained, the creation of a skin model with follicular units capable of generating an unpigmented hair shaft was accomplished.^83^ Here, micromolding was used to create, in fibroblast‐populated collagen I hydrogels, defined microwells in which DP spheroids were formed in situ. Keratinocytes were then seeded on top, engulfing the spheroids and differentiating into proper follicular layering over the control of the inductive DP cells. Hair shafts were only formed occasionally in these models thus, to further enhance DP cell inductivity, a subpopulation of LEF1 overexpressing DP cells was also used, significantly improving HF induction efficiency and the expression of hair lineage‐specific genes.

However, the most complete in vitro recreation of human HF achieved so far was developed entirely using pluripotent stem cell lines. In a quite distinct and successful approach, hair‐bearing skin organoids akin to 18‐week human fetal skin were produced. The formation of a cyst‐like skin organoid was attained from aggregates of pluripotent stem cells and using a step‐by‐step modulation of the TGF‐β, BMP, and FGF signaling pathways, to promote ectoderm and cranial‐neural crest induction.^162^ The formation of pigmented HFs within the skin organoids was observed after a 4/5‐month culture period. Importantly, the skin organoids also display a stratified epidermis and a dermis with SGs and neural innervation, linked to adjacent fat tissue. This study clearly demonstrates the relevance of selective modulation of hair development signaling pathways, as well as the advantage of reconstructing embryonic events guiding the natural development of the skin and associated appendages. Still, the long preparation time and associated costs represent considerable disadvantages comparably to the use of dissociated primary cells.

## 7 CONCLUSION AND FUTURE TRENDS

The need for human HF regenerative strategies, and in vitro models has been mostly nourished by the growing demonstration that murine and human skin and HFs are dissimilar.

The evidence has highlighted the importance of combining inductive DP cells and immature epithelial cells with a high proliferative capacity for the recreation of the HF cellular compartments, pushing efforts toward the maintenance of the trichogenic capacity of human cells in vitro. Even so, human hair regeneration promoted entirely from human adult cells is yet to be achieved. In truth, the literature concerning hair regeneration continues to be dominated by murine cell studies or chimeric human–murine combinations, instead of purely human approaches. On the other hand, poor success may also be partially explained by the lack of integrative approaches that go beyond the segregated exploitation of strategies to improve either the inductivity of DP cells or the competence of epithelial cells. In the future, comparative studies capable of identifying the most promising trichogenic combinations may allow significant developments in the field.

Meanwhile, other mesenchymal and epithelial human cell sources may be considered for HF regeneration. Human bone marrow or umbilical cord‐derived cells were able to induce HF formation after co‐transplantation with human ORS keratinocytes in mice and to promote hair bulb reformation in an ex vivo assay with transected human HFs.^163^ Furthermore, human adipose‐derived stem cells overexpressing PDGF‐A, Sox2, and β‐catenin showed higher ALP expression levels than the nontransfected counterparts or hDP cells^164^ thus representing a potential and easily available, mesenchymal cell source for HF reformulation. In an era of stem cell engineering, the use of iPSCs, from available and more easily reprogrammable autologous epithelial and mesenchymal sources, may also rapidly expand and led to important breakthroughs, such as the recently reported hair‐bearing skin organoids.^162^ Nonetheless, some constraints should be first overcome, such as the differentiation of the iPSCs toward the phenotypes of interest. We still have a long road ahead to achieve this goal, but the work by Lee et al.^162^ provide hope for iPSCs future applications in the HF regenerative field.

The most complex and functional HF models achieved so far comprise the whole skin with HF appendage reformation. Besides HF integration in a supportive biomimetic skin, the inclusion of other HF specialized cell populations, such as neuronal cells and melanocytes or melanocyte precursors in the models are necessary to bioengineer functional and aesthetically acceptable hairs. Also, the macroenvironment outside of the HF should be considered, particularly the adipose tissue given its regulatory role in hair development and growth. Mature HFs are deeply rooted in the dWAT and previous studies demonstrated that intradermal adipogenesis is intimately coupled with the HF stem cell activity and cycle progression.^165^ Immature adipocytes in the dWAT are necessary for follicular stem cell activation, while mature adipocytes promote follicular differentiation by inhibiting bulge activity,^166^ supporting the relevance of the adipose tissue for HF regenerative strategies.

Besides the cellular counterparts, the HF microenvironment constitutes, by itself, a challenge when bioengineering HFs. The ECM, in particular, has a key role in directing hair growth and the maintenance of cell function, which also varies with physiological HF cycling. Despite the knowledge generated on this subject, its translation has been limited and bioengineered HF models continue to be elementary and lacking an appropriate recreation of the complexity of the ECM or signaling molecules involved in hair control. Most strategies rely on the encapsulation of heterotypic hDP/epithelial cell combinations in ECM‐based hydrogels or their incorporation in the organotypic skin model; however, the adoption of advanced biofabrication techniques could improve our HF biomimicry capacity. The use of 3D‐bioprinting and associated layer‐by‐layer deposition of cells and respective ECM component, in particular, hold the promise to accurately recapitulate the 3D‐architecture and milieu of the HF, along with its cell–cell and cell–ECM interactions. This explains why commercial companies such as the L'Oreal/Poietis partnership or Organovo, Inc have announced their intention to bioprint HFs for in vitro testing applications. The latter even released a patent for the 3D‐bioprinting of HFs in recipient skin^167^ but failed to promote neohair formation. Nevertheless, the application of this addictive manufacturing technology by researchers working in the hair restoration field is still taking its first steps. In the future, successful outcomes will be intimately connected with the development of ECM biomimetic cell‐laden bioinks and the maintenance of the native properties of the cells in culture.

Ultimately, the techniques and knowledge generated when pursuing HF recreation in vitro also hold potential for regenerative purposes. Companies like RepliCel and HairClone have been working on the concept of rejuvenating miniaturized/resting follicles by, respectively, injecting autologous DS or DP cells in the patients' scalp to produce thicker terminal hair shafts. The former published a phase I/IIa study in patients with androgenic alopecia but, so far, they were only able to temporarily improve hair density and diameter.^168^ Alternatively, although only theoretical for now, the use of small amounts of donor HF samples to isolate, propagate and even genetically manipulate relevant cell populations in vitro would be ideal. These could then be used to promote HF de novo formation in situ, or to bioengineer HFs, allowing the production of ready‐to‐use follicular units that could be transferred back to the patient for hair restoration. Another important milestone would be the incorporation of HF‐like structures in skin substitutes, or the inclusion of cellular and/or bioactive clues that could sustain HF regeneration after implantation, also contributing to their functionality. Additionally, and depending on the cellular populations used and their stemness, a variable degree of self‐regeneration capacity could be granted to the skin substitutes, making them more likely to reach long‐term use and representing a more effective treatment for patients with severe skin damage.

Future research may even provide knowledge to reprogram the resident skin cells of the patient and recreate hair morphogenic events in situ. The complexity and spatiotemporal controlled nature of the HF signaling interactions imply that this type of intervention would most likely require the modulation of multiple signaling pathways and to be addressed in a timely manner, making it less likely to succeed compared to autologous cell‐based therapies. Nonetheless, there are conditions where simpler signaling modulation with inductive factors may prove sufficient to promote hair restoration or cycle reactivation in preexisting HFs. For example, hair miniaturization and loss, observed in common baldness, have been associated with a defective conversion of bulge stem cells into proliferative progenitor cells,^169^ representing a prime example of a condition that can eventually be reversed by pharmacological intervention. Also, the knowledge that during wound^92^ and increased β‐catenin activity^170^ neofollicle formation can occur from the IFE, and not from adjacent bulge stem cells, encourage further exploration of these mechanisms in humans.

New and more effective treatment options for hair loss disorders are a major target of the pharmaceutical and cosmetic industry. Similarly, skin substitutes with increased functionality have long been a pressing requirement for effective cutaneous wound healing. The work performed in the last years in the bioengineering of purely human HFs in vitro and, particularly in the knowledge regarding HF regeneration have paved the way for future approaches with a high potential to result in clinically applicable solutions.

## CONFLICT OF INTEREST

The authors declare no conflict of interest.

## AUTHOR CONTRIBUTIONS

**Carla M. Abreu**: Conceptualization; data curation; visualization; writing‐original draft; writing‐review and editing. **Alexandra P. Marques**: Conceptualization; funding acquisition; project administration; validation; visualization; writing‐review and editing.

### PEER REVIEW

The peer review history for this article is available at https://publons.com/publon/10.1002/btm2.10235.

## Data Availability

Data sharing not applicable to this article as no datasets were generated or analyzed during the current study.
